# 3D reconstruction in complex parenchymal sparing liver surgery

**DOI:** 10.1016/j.heliyon.2023.e13857

**Published:** 2023-02-21

**Authors:** Alessandro Michele Bonomi, Alessia Kersik, Greta Bracchetti, Christian Cotsoglou

**Affiliations:** a General Surgery Department, ASST-Vimercate, 20871, Vimercate, Italy; b University of Milan, Via Festa Del Perdono, 7, 20122, Milan, Italy

## Abstract

**Background:**

Prognosis of stage IV colorectal cancer is related to control of liver metastasis. As of now, surgery provides survival advantage for patients affected by resectable colorectal liver metastases (CRLM), with parenchymal sparing strategies representing the most accepted strategy {[1]. In this setting, 3D reconstruction programs represent the newest available technological leap to improve anatomical accuracy [2]. Despite being quite expensive, 3D models have proved themselves as helpful adjunctive tools to enhance pre-operative strategy [3] in complex liver procedures, even in the eyes of expert hepatobiliary surgeons [4].

**Methods:**

We present a video describing the practical use of a custom-made 3D model, acquired following specific quality criteria [2], for a case of bilateral CLRM after neoadjuvant chemotherapy.

**Results:**

In our reported case and as described in the video, pre-operative visualization of 3D reconstructions altered significantly the pre-operative surgical plan. First, following the principles of parenchymal sparing surgery, challenging atypical resections of metastatic lesions close to main vessels (right posterior branch of the portal vein, inferior vena cava) were preferred to anatomic resections/major hepatectomies, allowing the highest projected future liver remnant volume possible (up to 65%) amongst different available strategies. Secondly, the order of hepatic resections was planned to follow a decreasing degree of difficulty, in order minimize the effect of blood redistribution after previous resections during parenchymal dissection (thus starting from atypical resections close to main vessels, followed by anatomical resections and atypical resections of superficial resections). In addition, the availability of the 3D model in the operating room was crucial in the surgical field to guide safe surgical pathways, especially during atypical resections of lesions close to the main vessels: detection and navigation were further enhanced thanks to tools of augmented reality that allowed the surgeon to manipulate the 3D model through a touchless sensor in a dedicated screen in the operating room and to replicate a mirroring snapshot of the surgical field, without compromising sterility nor the surgical set-up. In the setting of these complex liver procedures, the application of 3D printed models has been described [4]; when available, 3D printed models, particularly useful in the pre-operative phase when explaining the procedure to patients and relatives, have been reported to have comparable significant impact, with feedback from expert hepatobiliary surgeons that is very similar to the one we are reporting in our experience [4].

**Conclusion:**

Routine use of 3D technology does not claim to revolutionize the world of traditional imaging but may be impactful in helping the surgeon visualize the anatomy of that specific individual in a dynamic and three-dimensional way that is similar to the surgical field, thus improving multidisciplinary preoperative planning and intraoperative navigation during complex liver surgery.

## Ethical considerations

The case report discussed received full written informed consent by the patient involved. He agreed for the publication of images or information related to his clinical history.

## Author contribution statement

All authors listed have significantly contributed to the investigation, development and writing of this article.

## Funding statement

This research did not receive any specific grant from funding agencies in the public, commercial, or not-for-profit sectors.

## Data availability statement

No data was used for the research described in the article.

## Declaration of competing interest

The authors declare that they have no known competing financial interests or personal relationships that could have appeared to influence the work reported in this paper.
